# Hiding in Plain Sight: Interplay between Staphylococcal Biofilms and Host Immunity

**DOI:** 10.3389/fimmu.2014.00037

**Published:** 2014-02-05

**Authors:** Tyler D. Scherr, Cortney E. Heim, John M. Morrison, Tammy Kielian

**Affiliations:** ^1^Department of Pathology and Microbiology, University of Nebraska Medical Center, Omaha, NE, USA

**Keywords:** *Staphylococcus aureus*, *Staphylococcus epidermidis*, biofilm, macrophage, neutrophil

## Abstract

*Staphylococcus aureus* and *Staphylococcus epidermidis* are notable for their propensity to form biofilms on implanted medical devices. Staphylococcal biofilm infections are typified by their recalcitrance to antibiotics and ability to circumvent host immune-mediated clearance, resulting in the establishment of chronic infections that are often recurrent in nature. Indeed, the immunomodulatory lifestyle of biofilms seemingly shapes the host immune response to ensure biofilm engraftment and persistence in an immune competent host. Here, we provide a brief review of the mechanisms whereby *S. aureus* and *S. epidermidis* biofilms manipulate host–pathogen interactions and discuss the concept of microenvironment maintenance in infectious outcomes, as well as speculate how these findings pertain to the challenges of staphylococcal vaccine development.

## Introduction

*Staphylococcus aureus* and *Staphylococcus epidermidis* are highly opportunistic pathogens and a major cause of nosocomial and community-associated infections (1–4). While both staphylococcal species harbor multiple virulence factors, they are also capable of biofilm formation, which represents a communal virulence determinant to circumvent immune-mediated clearance and establish persistent infection (5–8). Most medical device-associated biofilm infections are caused by *S. epidermidis* and *S. aureus*, and both species can also establish biofilms on native host surfaces, such as infective endocarditis and osteomyelitis (9–11). Biofilms are heterogeneous bacterial communities encased within a self-produced matrix composed of eDNA (12), proteins (13), and polysaccharides (14), and their composition is often dependent on environmental factors, such as nutrient availability and mechanical stress (15). Biofilms represent a spatially diverse population of cells in terms of gene expression and metabolic activity, which is thought to arise in response to differences in nutrient, pH, and oxygen gradients within the structure (5, 16). Biofilm infections are recalcitrant to antibiotics (17, 18), which often necessitates removal of the infected device or native tissue and is associated with significant morbidity and economic impact (10, 19, 20). Indeed, it has been estimated that approximately $1.8 billion is spent annually in the US for the treatment and clinical management of orthopedic implant-related infections (21, 22).

Neutrophils and macrophages are the main innate immune effectors against acute planktonic staphylococcal infections (i.e., abscess, sepsis) (23–29). Neutrophil antimicrobial activity is mediated by defensins, cathelicidins, and lysozymes, as well as reactive oxygen species catalyzed by NADPH oxidase (30, 31). Recently, neutrophil and macrophage extracellular traps (NETs and METs, respectively) have been identified as another means of antimicrobial action (31–34). This “beyond the grave” mechanism is typified by an extracellular net of DNA released from dying phagocytes that contains localized islands of lytic enzymes that kill ensnared extracellular bacteria. Classically activated (M1) macrophages are key microbicidal effectors that exert their effector functions, in part, through the production of reactive oxygen and nitrogen species, in addition to proinflammatory cytokines (35–38). Macrophages also clear extracellular bacteria by phagocytosis, which is tightly linked with proinflammatory cytokine and chemokine production to initiate adaptive immune responses (39–41). Collectively, these innate leukocyte responses, coupled with complement activation, usually ensure the successful clearance of planktonic staphylococcal infections in an immune competent host.

**Figure 1:**
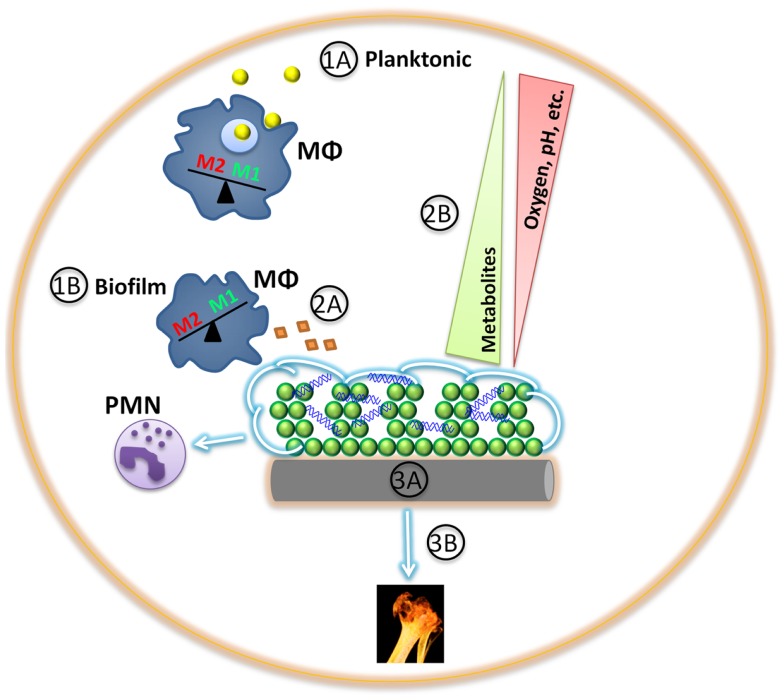
**Game of hide-and-seek between staphylococcal biofilms and host innate immunity**. **(1A)** Planktonic staphylococci (yellow) are more readily phagocytosed and cleared by innate immune cells, such as macrophages (MΦ; blue) and neutrophils (PMNs), triggering a M1 proinflammatory response. **(1B)** Differential gene expression between planktonic and biofilm staphylococci provide survival benefits in harsh environments, such as the presence of an extracellular matrix (green). In addition, *S. aureus* biofilms exhibit differential gene expression in response to macrophages or neutrophils (violet), shielding the biofilm from detection by the former while facilitating cytotoxicity of the latter. **(2A)** Staphylococcal biofilm protein secretion *in vivo* is relatively unknown and may represent fertile ground for clinical intervention and/or vaccine development. **(2B)** Staphylococcal biofilms are composed of a large biomass, of which some cells are metabolically active and transform the surrounding microenvironment by establishing various metabolic gradients. **(3A)** Implanted medical devices (gray) not only provide a scaffold for bacterial adhesion and biofilm formation that is facilitated through host protein deposition (tan), but can also induce a persistent, anti-inflammatory immune response that favors bacterial persistence. **(3B)**. Tissue damage provoked by staphylococcal nutrient procurement may further incite an overall anti-inflammatory milieu.

In contrast, a very different story has emerged regarding biofilm infections (Figure 1). Recent studies have demonstrated that staphylococcal biofilms actively skew host immunity toward an anti-inflammatory, pro-fibrotic response that favors bacterial persistence (6, 42–44). This is typified by alternatively activated (M2) macrophages and arginase-1 (Arg1) activity, resulting in urea and ornithine production, which are involved in collagen formation and tissue remodeling (35, 45, 46). Our laboratory has shown that macrophages associated with *S. aureus* biofilms both *in vitro* and *in vivo* have decreased inducible nitric oxide synthase (iNOS) concomitant with increased Arg1 expression, as well as attenuated cytokine and chemokine production (6, 43, 44). Similar findings have been reported in response to *S. epidermidis* biofilms (42, 47–49) and biofilms from other bacterial species (50–53), suggesting a conserved mechanism exists to thwart host immunity to ensure biofilm persistence (54). However, recent data suggest a protective role of anti-inflammatory Th2/Treg cells, as opposed to proinflammatory Th1/Th17 signaling, during early *S. aureus* biofilm development (55), highlighting the complexity of staphylococcal biofilm infections and perhaps distinctions between innate and adaptive immune mechanisms. For in-depth discussions on host immunity to staphylococcal infections, the reader is referred to recent reviews (7, 56–58). This Perspective is focused on how changes in biofilm gene expression/secretion impact host immune mechanisms and potential implications regarding novel therapeutic strategies.

## Differential Gene Expression in Staphylococcal Biofilms: A Game of Hide-and-Seek

Staphylococcal biofilms exhibit distinct gene expression profiles compared to planktonic cells, demonstrating unique growth differences between the two lifestyles (16, 59, 60). One major disparity in staphylococcal biofilms is the induction of genes associated with potential acid tolerance response pathways (i.e., arginine deiminase and urease), reflecting anaerobic growth and the need for pH homeostasis (5, 16). In addition, an increased demand for pyrimidine nucleotide biosynthesis has been associated with the biofilm lifestyle of staphylococci (16) and other bacterial species (61, 62). The DNA-binding regulatory protein SarA is critical for biofilm formation and virulence factor expression in both *S. aureus* and *S. epidermidis*. SarA acts, in part, through the accessory gene regulator *agr*, which encodes a quorum-sensing system responsible for transitioning the synthesis of surface and adhesive molecules to secreted factors and is an important regulatory switch between planktonic and biofilm lifestyles (63–66).

In addition to unique genetic changes during biofilm growth, we have recently reported that innate immune cells (i.e., macrophages and neutrophils) alter biofilm gene expression profiles (67). Transcriptome analysis of *S. aureus* biofilms following macrophage exposure revealed a rapid down-regulation of over 550 staphylococcal genes within a 1 h period. This effect occurred despite the fact that macrophages did not significantly invade the biofilm or exhibit phagocytic activity. This transcriptional repression was mitigated after 24 h, which was likely explained by the significant degree of macrophage death observed at this late interval. In essence, the biofilm was no longer required to cloak itself, since the threat of macrophage attack had dissipated. In contrast, although neutrophils displayed a much higher propensity for biofilm invasion and phagocytosis, they did not significantly alter the biofilm transcriptome over similar time periods (67). This data supports previous reports demonstrating that biofilms are not inherently protected from neutrophil assault (44, 68, 69).

These results suggest that staphylococcal biofilms “hide” in response to macrophages while they elect to tackle neutrophil challenge head on. But why does the biofilm down-regulate gene expression in the face of a seemingly inactive macrophage challenge and barely break a transcriptional sweat when confronted with an assault of phagocytic neutrophils? One possibility involves the reported ability of *S. aureus* to survive intracellularly in neutrophils (70, 71). Another is that *S. aureus* secretes a number of neutrophil-lytic toxins, including α-toxin (Hla) (72) and phenol-soluble modulins (PSMs) (73–77). Interestingly, both Hla and PSMs are under *agr* control, and *agr* expression was significantly increased in *S. aureus* biofilms following neutrophil exposure (67, 74). Notably, we have found few neutrophil infiltrates in either *S. aureus* catheter-associated or orthopedic implant models of biofilm infection *in vivo* (7, 44). Likewise, the exogenous introduction of neutrophils at the site of *S. aureus* biofilm infection is not capable of limiting bacterial growth, whereas M1-activated macrophages can significantly prevent or clear established biofilms (44). Collectively, these findings highlight the well-adapted nature of *S. aureus* biofilms to neutrophils. Although both neutrophils and macrophages are phagocytic, macrophages are recognized as a signaling hub during bacterial infections to coordinate evolving immune responses through cytokine and chemokine release (78–80). While it is possible that staphylococcal biofilms are more responsive to this secreted milieu than physical disruption via phagocytosis, it is clear that they respond differently to challenges from distinct leukocyte populations in regards to their transcriptional profile.

## Microenvironment Modulation

Previous work has characterized numerous secreted virulence factors used by staphylococci that target specific host cell populations. *S. aureus*, in particular, utilizes numerous hemolysins (81), leukocidins (82, 83), and proteases to evade the host immune system (84). While staphylococci certainly divert resources from secreted to structural proteins during early biofilm growth, protein secretion is still maintained in biofilms. For example, several reports have demonstrated that various bacterial biofilm-forming species secrete peptides and full-length proteins (i.e., Esp) that interfere with biofilm development of competing organisms, presumably to eliminate competition for a biofilm-friendly niche (85–87). Additionally, studies have demonstrated evidence of host-biofilm cross-talk involving the quorum-sensing molecules *N*-Acyl homoserine lactones (AHL) (88, 89). Therefore, it is likely that staphylococcal biofilms also secrete factors *in vivo*, perhaps under quorum-sensing system regulation, in an attempt to evade immune recognition and clearance. However, the identity of such molecules remains to be elucidated but could represent future attractive anti-biofilm agents.

Besides virulence factors, staphylococci also secrete molecules for nutrient procurement and cell signaling. For example, siderophores are critical for iron acquisition (90), while several signaling molecules are important for biofilm remodeling and dispersal, such as auto-inducing peptide (AIP) (63), nuclease (91), and PSMs (74). While the primary roles of these molecules are apparently unrelated to immune interactions, recent evidence suggests the potential for alternative functions. For example, nuclease, which mediates biofilm dispersal (65, 91), can also participate in NET degradation (32). Recently, nuclease in combination with adenosine synthase has been implicated in the conversion of NETs to leukotoxic deoxyadenosine, highlighting a clever means whereby *S. aureus* turns the immune response against itself (92). In addition, *S. aureus* PSM expression is induced following neutrophil phagocytosis, resulting in neutrophil lysis and bacterial escape (74, 76, 93). This process is regulated by the stringent response characterized by the synthesis of the intracellular signaling alarmone (p)ppGpp (93). Therefore, the stringent response provides yet another means of staphylococcal adaptation in response to select immune pressures, something that has previously been well-established in relation to nutrient availability and metabolism (94–99). Indeed, recent evidence has implicated (p)ppGpp and di-cyclic NTPs as critical signals in the switch from planktonic to biofilm growth (100).

However, secreted factors are not the only means whereby staphylococcal biofilms could regulate the host response in an exogenous manner. While bacteria have a plethora of environmental sensory mechanisms at their disposal, host immune cells are also very sensitive to their surrounding environment (101–103). Because biofilms represent a large biomass, the sum metabolic activity of the bacteria themselves would be expected to have an impact on pH and oxygen levels in the surrounding tissue microenvironment. Indeed, even subtle changes in pH (101, 104) or oxygen (102) can significantly alter the nature of the immune response. Furthermore, novel research with fungal and bacterial biofilms has identified a coordinated system of ROS signaling for biofilm maturation (105). It will be interesting to see what, if any, impact this biofilm ROS gradient has on the host immune response and whether host-generated ROS can also act as a signaling molecule within the biofilm to influence target pathways, such as biofilm formation via quorum-sensing (105).

## Hijacking the Host Response

Most implanted medical devices are at risk for biofilm colonization (4, 10, 11, 15). Upon placement, the device is conditioned by host proteins, such as fibronectin and collagen, which can serve as ligands for bacterial attachment via microbial surface components recognizing adhesive matrix molecules (MSCRAMMS) (4, 9, 11, 15, 106, 107). Therefore, the wound healing responses elicited after medical device implantation can inadvertently provide a rich environment for bacterial colonization; however, they can also set the stage for a very specific immune response that is incapable of mediating bacterial clearance, as described below (108). Staphylococcal biofilm-associated protein (Bap) can facilitate adherence to host epithelial cells through the inhibition of MSCRAMM-mediated attachment (109, 110). Following adherence, bacteria begin to proliferate and accumulate by producing adherence factors and matrix components, resulting in biofilm formation. Bacterial-derived accumulation-associated protein (Aap) and polysaccharide intercellular adhesin (PIA) are important accumulation molecules in *S. epidermidis*.

Our laboratory has reported that *S. aureus* biofilms promote their growth and expansion by manipulating the host immune response (6, 43, 44, 111). Several studies demonstrated that staphylococci have developed strategies to interfere with M1 macrophage polarization either by directly inhibiting antimicrobial activity or hindering M1 cytokine production (6, 42, 44, 48). In addition, the expression of numerous proinflammatory mediators is significantly attenuated during *S. aureus* biofilm infection (6, 7, 43, 44). Since many of these molecules are also associated with wound healing responses, their active repression by staphylococcal biofilms is likely one mechanism to explain the paucity of neutrophil and M1-activated macrophage infiltrates into biofilms *in vivo* (6, 43, 44).

Besides dampening the local inflammatory environment, numerous mechanisms likely exist to explain the chronicity of staphylococcal biofilm infections in an immune competent host. For example, we have demonstrated the preferential accumulation of M2-activated macrophages in *S. aureus* biofilms *in vivo* and *in vitro* typified by Arg1 concomitant with minimal iNOS expression (6, 43, 44). Increased Arg1 activity during staphylococcal infection can regulate T cell proliferation and effector functions via arginine depletion from the environment, resulting in defective TCR signaling from inhibiting CD3ζ expression, cell cycle, and cytokine production (112–116). Indeed, our laboratory has found few T cell infiltrates associated with *S. aureus* biofilm infections, whereas another group reported Th2/Treg involvement in biofilm clearance (7, 55). The reasons for these discrepancies are not clear but may arise from differences in experimental models and/or *S. aureus* strains tested. Impaired adaptive immunity or memory responses could compromise the success of vaccine strategies against staphylococcal biofilms.

## Future Directions

As ongoing staphylococcal biofilm research elucidates mechanisms contributing to the persistence and virulence of these infections, it is becoming clear that novel strategies are needed to combat the complex biofilm lifestyle. The inherent ability of *S. aureus* to circumvent immune-mediated clearance has complicated staphylococcal vaccine development. Indeed, previous studies have shown that *S. aureus* infection alone is not sufficient to induce protective immunity (117). Moreover, recent vaccine development efforts have stalled in clinical trials, likely due to the organism’s immunomodulatory capabilities coupled with the differential expression of targeted antigens *in vivo* (118–120). Another challenge is to develop a vaccine efficacious against both planktonic and biofilm infections that provides protection across a wide range of *S. aureus* clonal complex groups (121).

Recent staphylococcal vaccine efforts have utilized new approaches to increase efficacy. Vaccination with attenuated *S. aureus* strains (122) has led to the discovery of protective antigens with vaccine potential in a process termed “genetic vaccinology” (123, 124). In addition, multivalent vaccines targeting multiple secreted and cell surface-associated virulence factors from both planktonic (125), and biofilm (126, 127) modes of growth have been employed with some success *in vivo* (121, 128). Furthermore, the use of Toll-like receptor agonists as adjuvants and novel DNA vaccines are currently under investigation (129, 130). While the efforts of our laboratory and others have implicated a subset of putative factors responsible for the immunosuppressive and chronic nature of staphylococcal biofilms, significant obstacles remain for developing a successful vaccine. In our opinion, future anti-staphylococcal therapeutics for the biofilm lifestyle will likely require a combinatorial approach of bacteriocidal and immunostimulatory treatments.

In this context, a successful staphylococcal vaccine may be facilitated by a more comprehensive understanding of the staphylococcal biofilm transcriptome and secretome, particularly in response to relevant host phagocytes, which will be an important next step toward replicating complex *in vivo* interactions. Additional insights into staphylococcal biofilm metabolism, in particular, how the biofilm metabolome changes once immune cells are encountered, may be beneficial as it could facilitate the identification of virulence determinants for vaccine development. A recent analogy is the use of metabolic stimuli to induce aminoglycoside susceptibility in otherwise recalcitrant *S. aureus* cell populations, such as biofilms (131). Also, characterizing how the natural wound healing response promotes bacterial colonization could provide invaluable information on key immune cell populations for vaccine targeting. A better understanding of how biofilms manipulate the host immune response should provide valuable insights to direct the continued evolution of staphylococcal vaccines. Until, we better define how staphylococci circumvent host immunity, they will likely continue to have the upper hand.

## Conflict of Interest Statement

The authors declare that the research was conducted in the absence of any commercial or financial relationships that could be construed as a potential conflict of interest.
